# Cubes to Cubes: Organization of MgO Particles into One-Dimensional and Two-Dimensional Nanostructures

**DOI:** 10.1021/acs.cgd.1c00535

**Published:** 2021-07-02

**Authors:** Daniel Thomele, Stefan O. Baumann, Johannes Schneider, Andreas K. Sternig, Sarah Shulda, Ryan M. Richards, Thomas Schwab, Gregor A. Zickler, Gilles R. Bourret, Oliver Diwald

**Affiliations:** † Department of Chemistry and Physics of Materials, Paris-Lodron University Salzburg, Jakob Haringerstrasse 2a, Salzburg, 5020, Austria; ‡ Institute of Particle Technology (LFG), Friedrich-Alexander-Universität Erlangen-Nürnberg, Cauerstraße 4, Erlangen, 91058, Germany; § Department of Chemistry, Colorado School of Mines, Golden, Colorado 80401, United States

## Abstract

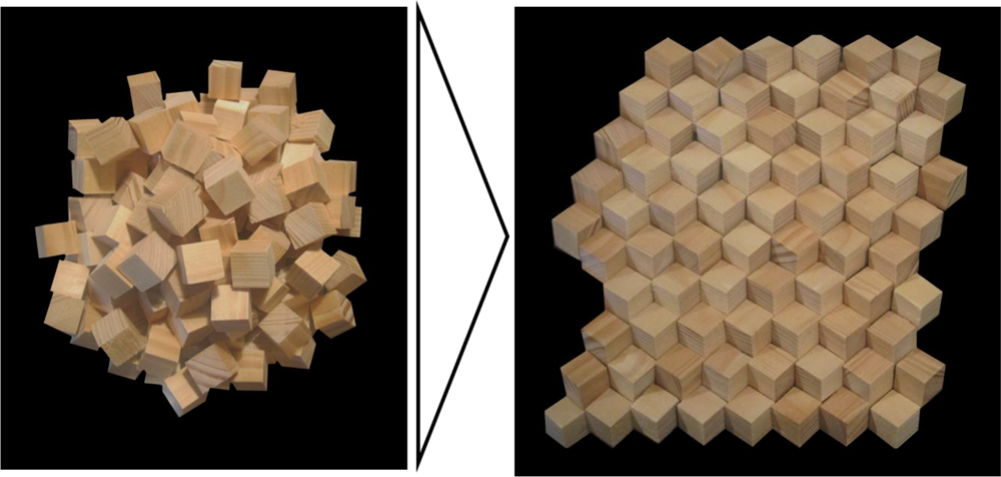

Developing simple, inexpensive, and environmentally benign approaches to integrate morphologically well-defined nanoscale building blocks into larger high surface area materials is a key challenge in materials design and processing. In this work, we investigate the fundamental surface phenomena between MgO and water (both adsorption and desorption) with particles prepared via a vapor-phase process (MgO nanocubes) and a modified aerogel process (MgO(111) nanosheets). Through these studies, we unravel a strategy to assemble individual MgO nanoparticles into extended faceted single-crystalline MgO nanosheets and nanorods with well-defined exposed surfaces and edges. This reorganization can be triggered by the presence of H_2_O vapor or bulk liquid water. Water adsorption and the progressive conversion of vapor-phase grown oxide particles into hydroxides give rise to either one-dimensional or two-dimensional (1D or 2D) structures of high dispersion and surface area. The resulting Mg(OH)_2_ lamella with a predominant (001) surface termination are well-suited precursor structures for their topotactic conversion into laterally extended and uniform MgO(111) grain surface configurations. To understand the potential of polar (111) surfaces for faceting and surface reconstruction effects associated with water desorption, we investigated the stability of MgO(111) nanosheets during vacuum annealing and electron beam exposure. The significant surface reconstruction of the MgO(111) surfaces observed shows that adsorbate-free (111)-terminated surfaces of unsupported MgO nanostructures reconstruct rather than remain as charged planes of either three-fold coordinated O^2–^ ion or Mg^2+^ ions. Thus, here we demonstrate the role water can play in surface formation and reconstruction by bridging wet chemical and surface science inspired approaches.

## Abstract 2

The organization of MgO nanocubes as primary building blocks can be achieved by materials contact with water and subsequent thermal treatment in a vacuum.

## Introduction

It is well established that the size, shape, and faceting of metal oxide particles have a substantial impact on their catalytic activity as well as their chemical properties when utilized as catalyst supports. Realizing cost-effective and environmentally benign approaches to controllably incorporate metal oxide nanoparticles into extended structures with well-defined faceting would be a significant step in progressing materials development across numerous fields of research and industrial applications. One promising approach, presented herein, is the topotactic rearrangement of metal oxide nanoparticles after their exposure to aqueous conditions.

The transformation behavior of metal oxide particles in aqueous environments is characterized by a variety of interaction pathways that include oriented attachment and dissolution–recrystallization processes.^1−5^ In that regard, particles with characteristic and defined crystal habits are promising building units to generate spatially organized nanostructures composed of uniformly sized and shaped surface elements. As a result of their simple morphology and the limited number of characteristic local surface structures, cubic metal oxide particles with a rock salt structure (such as MgO, CaO, CoO, MnO, NiO, and FeO) represent particularly well-suited model compounds to study such phenomena.

This is especially true for MgO particles produced by gas phase synthesis techniques such as chemical vapor synthesis (CVS),^6^ flame spray pyrolysis (FSP),^7^ or simply the combustion of metallic magnesium in air.^6^ When these MgO particles are synthesized at high temperatures and in anhydrous oxygen atmosphere, they adopt a typical cubic morphology, bound by the thermodynamically most stable surfaces.^8^ Such particles have well-defined crystallinity, crystallite habit, particle size distribution,^7,9^ and host a high abundance of characteristic surface features such as corners, edges, and step edges as well as other defects which can be identified by different structural characterizations (XRD, TEM) and spectroscopic (FT-IR, UV–vis diffuse reflectance, photoluminescence)^10−12^ techniques. In addition, it has been demonstrated that water adsorption and surface hydroxylation have a strong influence on the surface morphology of MgO grains.^13^ Ab-initio calculations have shown that the order of surface energies of the different (100), (110), and (111) planes is reversed as a consequence of the stronger adsorption (chemisorption) energy of water to the high index planes.^8,13−15^ Protonation stabilizes the otherwise unstable (111) surface, which exhibits the highest stability under ambient conditions.^16,17^ Thus, cubic MgO is well-suited to study processes that rely on interfacial and growth phenomena within metal oxide particles when exposed to an aqueous environment.^18^

**Figure 1:**
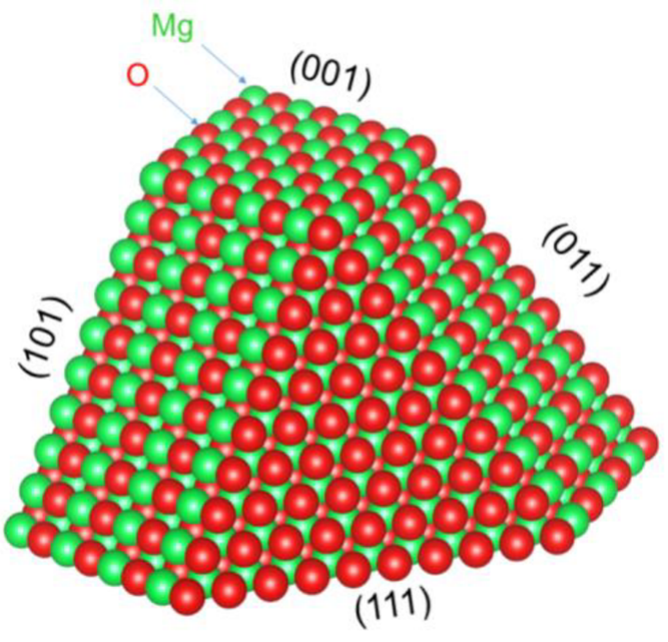
A prototypical rock salt structured metal oxide, MgO, with the (100), (110), and (111) facets shown. The (100) and (110) facets are composed of alternating cations and anions, while the (111) facet is made up of a single type of ion. It consists either exclusively of cations or of anions.

The surface structure, and hence chemistry, of metal oxides is in large part dependent on the oxide facet. Of particular interest is the (111) facet of MgO, which has demonstrated increased activity for varying applications including heterogeneous catalysis^19^ and gas sorption.^20^ The rock salt (111) surface is classified as a Tasker “type 3” surface with alternating layers of cations and anions (Figure 1). According to Tasker, such a surface, if bulk-terminated, would be unstable: the alternating layers of cations and anions would create a diverging electric field with an infinite surface energy.^21^ Therefore, charge compensation must occur to alleviate the infinite surface dipole. While there are many possible modes to stabilize a Tasker 3 surface, theory points to three primary scenarios: surface reconstruction, electronic relaxation, and adsorption of species that stabilize the electric field.^22−24^ Stabilization can also change depending on the nanoscale properties of the material.^25,26^ Through some mode of stabilization, several stabilized Tasker 3 surfaces have been synthesized (e.g., single crystals,^27−29^ films,^25,30−32^ nanowires,^23^ nanocrystals),^23,33−36^ and they demonstrate properties significantly different than those of the more typical (100) and (110) surfaces of the same material,^35−38^ with enhanced activity being attributed to under-coordinated sites at corners and step terraces, as well as exposed O^2–^ sites at point defects. Recently, it was reported that MgO(111) treated at 800 °C demonstrated a 65% increase in CO_2_ capacity despite suffering from a 30% decrease in surface area due to sintering. It was established that the heat treatment removed surface hydrogen exposing the low-coordinated O^2–^ necessary for CO_2_ sorption.^20^ In other recent work, Gates and Richards et al. succeeded in the uniform anchoring of iridium atoms over the edge and corner elements of such faceted MgO(111) microplanes to generate precise and periodic structures of atomically dispersed metals on a crystalline high surface area support.^39^

To take advantage of such surface-induced effects, a variety of solution-based syntheses have been developed to produce sheet-like MgO nanostructures with exposed {111} planes.^35,40^ Herein, we report on extended faceted single-crystalline MgO nanosheets and nanorods with well-defined exposed surfaces and edges synthesized via topotactic decomposition of sheets of hydroxylated MgO and Mg(OH)_2_. Such structures are of great interest because of the uniform distribution of their corner and edge elements and high degree of (111) faceting.

In the first part of this study, we report on the structural reorganization pathways that MgO nanocubes can undergo in the presence of H_2_O vapor or in contact with condensed water to generate characteristic 1D structures and 2D faceted surfaces. Vacuum annealing experiments at 273, 473, and 1173 K lead to different degrees of faceting, which can be explained by the different adsorbate coverages resulting from these different annealing temperatures. Additionally, we address the effect of reconstruction and faceting^41^ that occur at MgO(111) surfaces to compensate for the energetically unfavorable surface polarity that arises in the final stages of dehydroxylation and dehydration. Adsorbate removal can be achieved under high vacuum annealing at *T* > 1073 K or during electron beam irradiation within a transmission electron microscope. The transformation steps described here are simple and describe the conversion of nanocubes randomly organized within dry powders into regular stacks of nanocubes that are interconnected via the cube edges. The conversion process exclusively involves MgO nanoparticles, gaseous, or liquid water at defined dosages and subsequent vacuum treatment. Thus, we report a robust and simple route for the synthesis of ultrathin and thermally stable high surface area supports, which are in high-demand for numerous applications including heterogeneous catalysis. In the second part of this study, we discuss wet-chemistry derived MgO (111) nanosheets, stabilized by residual synthesis related adsorbates together with their structural derivatives that emerge upon vacuum annealing at *T* > 1073 K or extended electron beam exposure. Our results show that bare MgO(111) reconstructs into the more stable (100) surfaces,^41^ suggesting that completely bare polar MgO(111) surfaces do not exist.

## Experimental Methods

### Synthesis of MgO Nanocubes

For the production of MgO nanocubes, we use chemical vapor synthesis, which allows for the controlled evaporation and subsequent oxidation of alkaline earth metals under reduced pressures.^6,7^ Stable process conditions are guaranteed by spatially separating the evaporation and oxidation zone. The synthesis reactor consists of two quartz glass tubes inside a cylindrical furnace. The inner tube hosts ceramic ships with Mg pieces (99.98%, Aldrich), which are heated to 913 K assuring a metal vapor pressure of 1 mmHg column (1.33 mbar). An inert argon stream carries the metal vapor away from the evaporation zone to the end of the inner glass tube. There the Ar/metal vapor mixture encounters the oxidizing agent (O_2_), which is flowing through the outer glass tube. The exothermic oxidation reaction leads to a bright stable flame in the reactor, and MgO nanoparticles form because of the homogeneous nucleation and crystal growth in the gas phase. Thanks to continuous pumping, the residence time of nuclei within the flame remains short enough to prevent substantial coarsening and coalescence. A bypass system allows avoiding particle collection during uncontrolled process conditions, i.e., the heating and cooling phase. The total pressure in the CVS reactor is kept constant at 50 ± 3 mbar over the entire production process.

After the gas phase synthesis, the MgO nanoparticle powders are transferred into quartz glass cells, which allow one to carry out thermal activation of the powders in defined gas atmospheres. The as-obtained MgO powders are cleaned of organic contaminants by heating to 1123 K at a rate of 5 K·min^–1^ and exposure to molecular oxygen at this temperature. Then, the sample temperature was raised to 1173 K at pressures *p* < 5 × 10^–6^ mbar and kept at this temperature for 1 h until full dehydroxylation of the sample surface was achieved.^42^

After vacuum annealing, 50 mg of the MgO powder is dispersed in 100 mL of high-grade water (Millipore Simplicity M 185). The dispersion is stirred for 30 min on a magnetic stirrer. Alternatively and for control experiments that should rule out that CO_2_ uptake from the atmosphere could contribute to the here observed transformations, we also performed Ar flushing for convective mixing of the dispersion. Finally, the dispersion is centrifuged, and the solid material is dried using a membrane pump (*p* < 2 mbar) for 24 h prior to materials characterization.

The wet chemical synthesis of MgO(111) was first reported by the Richards group via a modified aerogel method.^35^ 4-Methoxybenzyl alcohol (also generically termed benzyl alcohol herein) is added as a directing agent and is hypothesized to interact with the hydroxyl group of the intermediate Mg(OH)-(OCH_3_) more strongly than methanol due to higher acidity, to form a material with a predominantly (111) surface. In the absence of the benzyl alcohol directing agent, (111)-oriented nanosheets were not observed. The addition of water induces hydrolysis and the resulting white sol–gel is then transferred to an autoclave reactor where it is purged with argon and then pressurized to 10 bar before heating to 265 °C. Upon heating, the pressure in the reactor increases to reach a pseudo-supercritical state where it is maintained. Pseudo-supercritical drying is performed by releasing the pressure while still hot, resulting in the dry white powder precursor, Mg(OH)_*x*_(OCH_3_)_2–*x*_. Calcination in air at 500 °C removes all carbon species, and hydroxyl-terminated MgO(111) nanosheets are obtained.

### Materials Characterization

X-ray diffraction (XRD) measurements were performed on a Bruker AXS D8 Advance diffractometer using Cu K_α_ radiation (λ = 154 pm). Specific surface areas were determined from nitrogen sorption isotherms acquired at 77 K (Micromeritics ASAP 2020). Diffractograms were recorded in time intervals of 9 min. Scanning electron microscopy (SEM) measurements were performed on a Zeiss Gemini Ultra 55 microscope operating at 20 kV. The transmission electron microscopy (TEM) investigations were performed on a Phillips CM300 UT operated at 300 kV for all samples except the MgO (111) pristine and annealed samples, which were imaged with a cold field emission gun JEOL JEM-F200 TEM at 200 kV. The electron dose received by the samples during the electron beam irradiation experiments was estimated from in situ measurement of the electron dose on the fluorescent screen. The TEM samples were prepared by casting small amounts of the dried metal oxide powders on the carbon grid.

## Results and Discussion

### MgO Nanocube Powder Exposure to Water Vapor and Dehydroxylation

**Figure 2:**
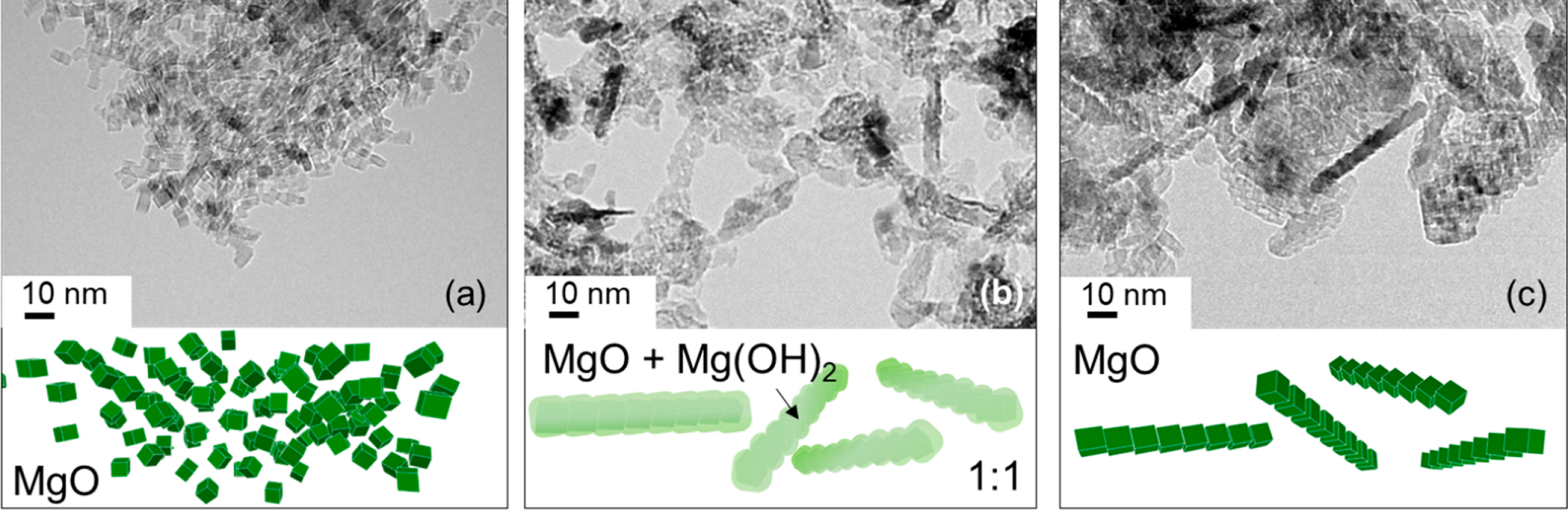
TEM images of MgO nanocubes: (a) after vacuum annealing of the powder, (b) after exposure to H_2_O vapor at *T* = 298 K and for 120 min (pH_2_O = 30 mbar) and (c) after reannealing of the material shown in (b) at *T* = 1173 K. On the basis of XRD data (see below Figure 5), the sample in (a) and (c) is pure MgO with average crystallite domains sizes below 10 nm, while the sample shown in (b) is composed of a 1:1 mixture of MgO (periclase) and Mg(OH)_2_ (brucite), as determined by Rietveld refinement (Supporting Information S1).

The interaction between MgO particles and water is manifold and leads to different structures depending on the concentration of water and its form of admission to the precleaned particle surfaces (i.e., contact with water vapor or immersion into a condensed bulk liquid). At partial pressures in the 1–30 mbar range, which is comparable to those in air, water adsorption leads to coverages of a few layers,^5,43^ and the dissociative adsorption of water results in surface energy changes that can trigger the formation of MgO nanocube stacking. Indeed, water vapor exposure of a MgO nanocube powder sample that was previously outgassed at *T* = 1173 K and at *p* < 10^–5^ mbar (Figure 2a) has a profound impact on the microstructure of the powder. The water vapor exposure was performed in a pre-evacuated closed system that guaranteed the exclusion of CO_2_, O_2_, and other impurities from the gas phase that may affect the dissolution–recrystallization processes described below. After 120 min of contact time with H_2_O (p(H_2_O) = 30 mbar), the samples contain a large number of elongated structures with widths that are typically larger than the size of the MgO nanoparticles prior to H_2_O contact. The image in Figure 2b reveals that these linear particle aggregates are morphologically less-defined as compared to the vacuum-annealed MgO particle systems (Figure 2a,c). This leads to slightly blurred and low-contrast bright-field TEM images, which typically change upon prolonged electron exposure during the TEM measurements (see below).

The original MgO building blocks that are present in these aggregates are covered with a characteristic shell of amorphous and crystalline Mg(OH)_2_, which emerges upon water adsorption and surface hydroxylation, as evidenced by XRD.^44^ XRD pattern analysis and Rietveld refinement of the data obtained on the samples presented in Figure 2b revealed a 1:1 phase mixture of MgO:Mg(OH)_2_ (Supporting Information, TEM Figure S1a,b and XRD, Figure S1c).

In these experiments, water vapor serves as an ultrapure hydroxylation agent for particle powders with precleaned surfaces. During the early stages of their hydroxylation and hydration, MgO nanocubes self-assemble into one-dimensional structures.^18^ Instead of stacking into straight bars, which would be the most effective way to reduce their surface area, we observed regularly displaced stacks of MgO particles, appearing as staggered particle ensembles.^18^ Previous DFT calculations pointed to the adsorption of water in different stages and analyzed the impact of water adsorption on the total energy of the MgO stack: (1) Water dissociation leads to the decoration of corners, edges, and ledges with surface hydroxyls; (2) water adsorption at terraces enables the formation of hydrogen-bonded networks that connect to the edges and corners; (3) further water adsorption leads to the coverage of remaining surfaces and to multilayer formation. The energetic stabilization of the displaced stacks over the straight bars arises from water adsorption at low-coordinated sites in combination with the decoration with MgO nanocube ledges and residual available terrace sites.^18^ As a result, the staggered MgO nanocube bar, which forms during the early stages of hydration, serves later as a structural backbone for the progressive conversion of the oxide into the hydroxide.

The changes in particle volume (compare Figure 2a,b) observed here are attributed to swelling effects that were already observed on larger MgO smoke cubes^45^ upon contact with condensed water and which partly originates from the larger specific volume of Mg(OH)_2_. Vacuum annealing at base pressures of *p* < 10^–5^ mbar and *T* = 1173 K reconverts all hydroxides into oxides as evidenced by XRD.^18^ Previous FT-IR experiments performed under comparable experimental conditions and on identical materials revealed completed dehydroxylation of the oxide surfaces. Elimination of the thermally most stable free OH groups with their characteristic absorptions bands at *ṽ* > 3700 cm^–1^ at *T* > 1100 K and *p* < 10^–5^ mbar provides strong evidence for the generation of adsorbate-free particle surfaces.^42,46,47^ We expect that similar decomposition,^48^ dehydration, and dehydroxylation processes^42^ are induced by electron-beam heating inside the vacuum chamber of the TEM instrument (see the experiments on MgO(111) nanosheets described below). As a result, vacuum-annealed MgO nanocubes previously exposed to water vapor contain characteristic elongated bar-like structures (Figure 2c) composed of staggered MgO nanocubes without any further evidence for (111) plane formation or faceting.

### MgO Nanocube Immersion into Condensed Water and Dehydroxylation

**Figure 3:**
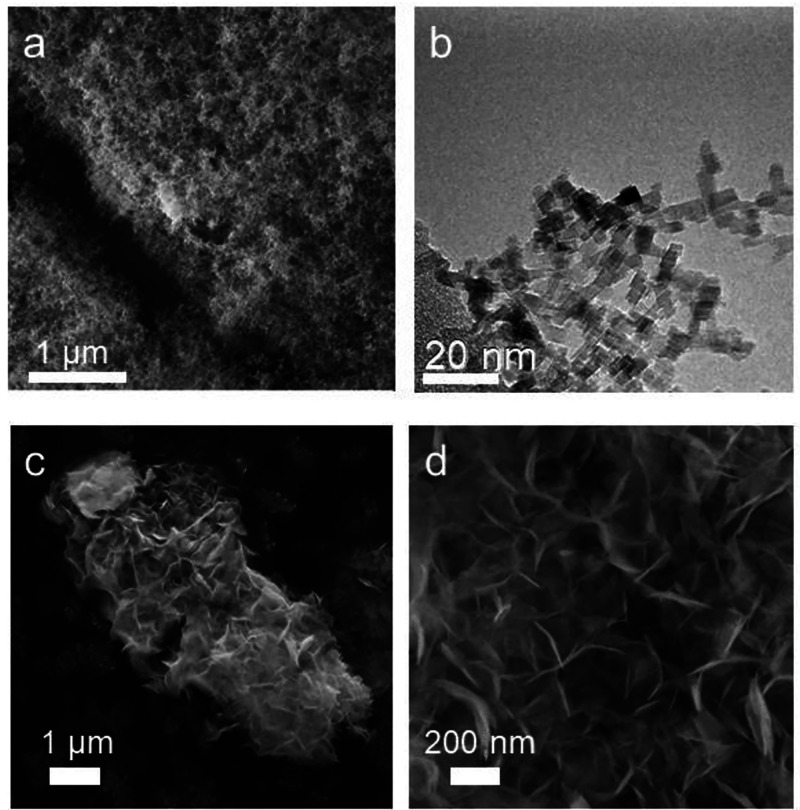
Scanning electron microscopy (a, c, and d) and transmission electron microscopy (b) images of MgO nanocube powder (a, b) as a starting material and dried Mg(OH)_2_ (c, d) after dissolution–recrystallization in pure water.

Alternatively, we generated aqueous Mg(OH)_2_ dispersions by particle immersion into liquid water in an argon atmosphere. Within the first minutes of MgO dissolution, the pH value of the supernatant solution increased from 6 to 10 and remained constant thereafter. Ar flushing was used for convective mixing. MgO nanocrystal dissolution and Mg(OH)_2_ recrystallization give rise to ultrathin sheets with high specific surface area (Figure 3c,d). The Mg(OH)_2_ microstructures imaged by SEM were analyzed after subsequent water removal and vacuum drying at room temperature. As a result, the MgO nanocube powder has been transformed into nest-like aggregates of thin nanosheets.

**Figure 4:**
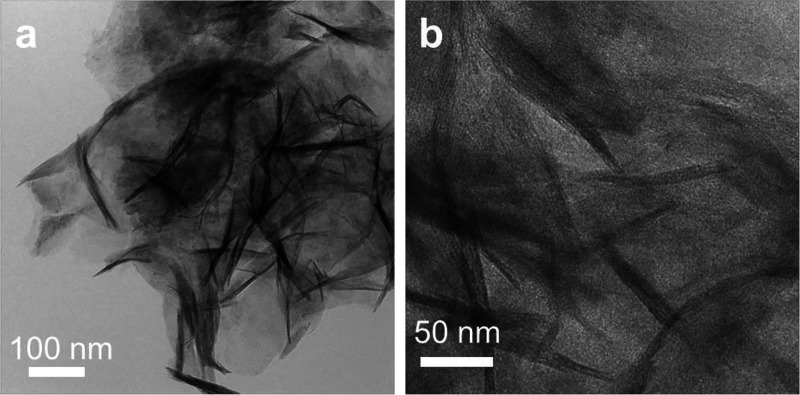
Transmission electron micrographs of Mg(OH)_2_ nanosheets isolated after dissolution of MgO nanocubes in pure water and subsequent water removal at room temperature. Images in (a) and (b) were taken at two different sample regions and at different magnifications.

These are a few nanometers thick and, based on the TEM data (Figure 4), can be described as Mg(OH)_2_ lamella that coexist with particles of needle-like habit that are attributed to scrolled-up Mg(OH)_2_ sheets.^6^

**Figure 5:**
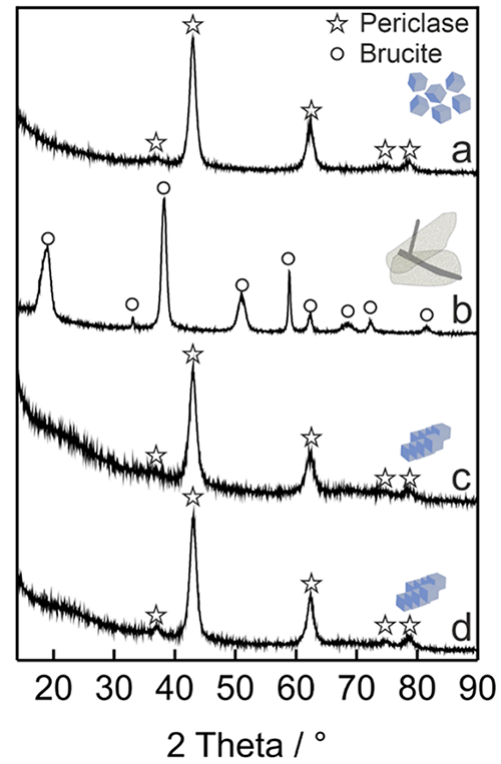
X-ray diffraction (XRD) patterns of (a) MgO nanocubes, (b) after dispersion in water and drying at RT (b) and vacuum annealing (*p* < 5 × 10^–6^ mbar) to 473 K (c) and 1173 K (d).

We acquired XRD pattern on the samples, before and after immersion into condensed water (Figure 5a,b, respectively). The diffraction data clearly show that a complete phase transformation into Mg(OH)_2_ has occurred in liquid water (Figure 5b). Using the basal (001) and the nonbasal (110) reflections of Mg(OH)_2_ at Bragg angles 2θ = 18.8° and 58.9° respectively, the following values for the crystallite domain sizes along the *a*-axis (*x*_001_) and the *c* axis (*x*_110_) were determined using the Scherrer equation: *x*_001_ = 3 nm and *x*_110_ = 20 nm, respectively, which are in reasonable agreement with the sheet-like morphology of the product structures observed by TEM. These, in turn, are well-suited precursor structures for the topotactic decomposition of the hydroxide into MgO.^48^

**Figure 6:**
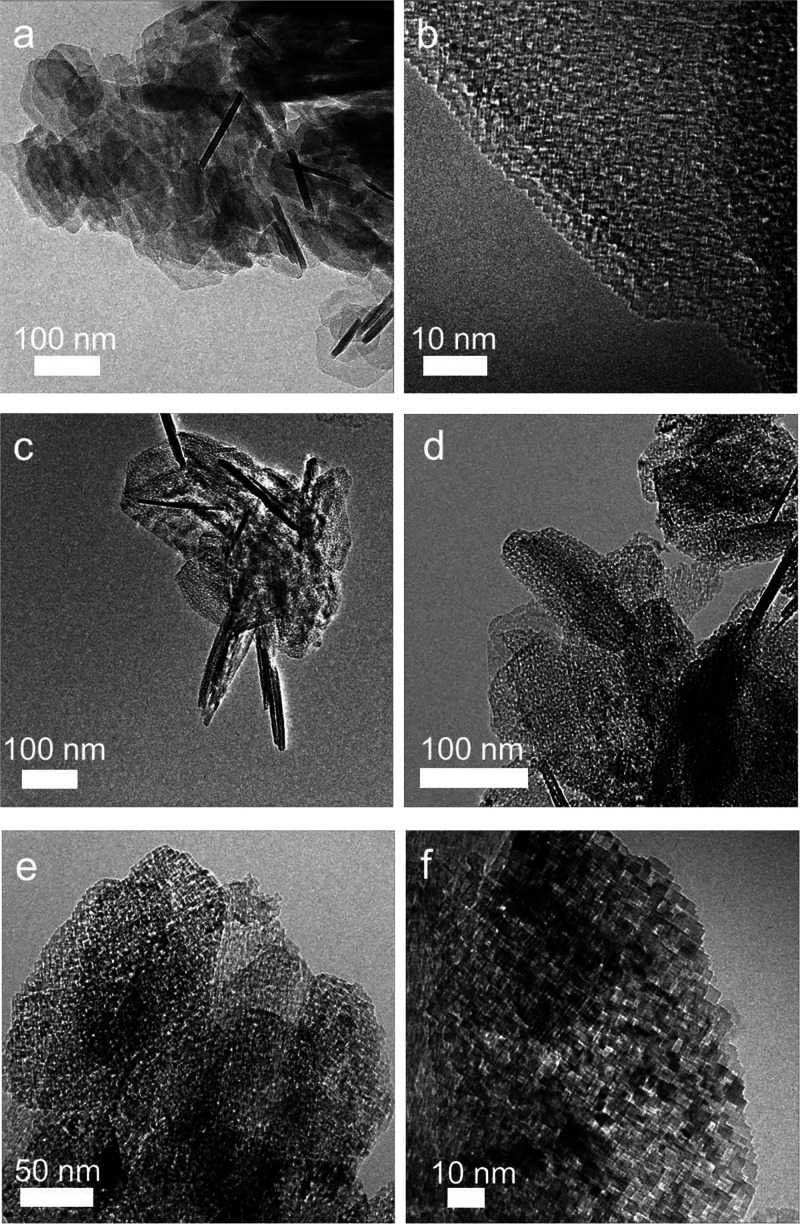
Transmission electron micrographs of MgO structures after vacuum annealing of Mg(OH)_2_ to 473 K (a and b) and to 1173 K (c–f).

Vacuum annealing (*p* < 10^–5^ mbar) at temperatures as low as 473 K reconverts the hydroxide into the oxide (Figure 5b,c) with a XRD pattern that is similar to that of the MgO sample after vacuum annealing and complete dehydroxylation (Figure 5a)^42^ at higher temperatures such as *T* ≥ 1173 K (Figures 5d and 6c–f). XRD analysis shows that the MgO cubelets have the same average crystallite domain size as the starting material, i.e., 7 ±1 nm (Figure 2a).^7^

TEM analysis of the vacuum -annealed samples revealed two characteristic morphological features: (a) elongated structures with lengths up to 400 nm. These are attributed to partially hydroxylated stacks of staggered MgO cubes.^18^ (b) extended plate-like grains that partially retained the shape of the parent hydroxide flakes (Figure 6c,d). They are made up of aggregated MgO nanocubes that exhibit MgO (100) microfacets^9,49^ and are connected by their edges after annealing at 1173 K. In terms of edge length, these cubes adopt sizes that are similar to the size of vapor-phase grown nanocubes, which is consistent with both (i) the values of the average crystallite domain size and (ii) the high specific surface areas measured by sorption analysis (Table 1).

**Table 1:** Average Nanocrystal Sizes Determined Using the Scherrer Equation (*d*_XRD_), Specific Surface Area Calculated from Average Nanocrystal Sizes (*S*_XRD_), Specific Surface Area Determined from N_2_ Adsorption (*S*_BET_), and Corresponding Average Particle Size (*d*_BET_) for MgO Nanocubes, Those after Dispersion in Water Followed by Drying in a Vacuum and after Vacuum Annealing at *p* < 5 × 10^–6^ mbar to 473 and 1173 K

	MgO	MgO + H_2_O	MgO 473 K	MgO 1173 K
*d*_XRD_/nm	6.4 ± 1.0	(2.9 × 17) ± 1.0^a^	7.0 ± 1.0	6.9 ± 1.0
*S*_XRD_/m^2^ g^–1^	262 ± 26	71 ± 7^b^	239 ± 24	243 ± 24
*S*_BET_/m^2^ g^–1^	296 ± 29	109 ± 10	103 ± 10	236 ± 23
*d*_BET_/nm	5.7 ± 1.0	15.4 ± 1.0	16.2 ± 1.0	7.1 ± 1.0
*V*_Pore_/cm^3^ g^–1^	1.14	1.25	0.93	1.00

a Evaluation of XRD patterns reveal an anisotropic crystallite domain size.

b Calculated under the assumption of rectangular shape crystalline domains with 17 nm in the *x*- and *y*-direction and 2.9 nm in the *z*-direction.

The transformation of Mg(OH)_2_, which was obtained by MgO dissolution in liquid water, into MgO corresponds to a topotactic Mg(OH)_2_ → MgO fragmentation process of the parallel hydroxide lamella into parallel polycrystalline metal oxide plates (Figure 6a).^9,48−53^ These results align with a recent environmental-cell (E-cell) dynamic high resolution transmission electron microscopy (D-HR-TEM) study^52^ that revealed atomic level details of such hydroxide decomposition reactions and characterized the lamellar nucleation and growth processes that generate host layer bending and local elastic strain. The resulting strain induces cracking and delamination at the nanometer level to generate these characteristic surface topologies of interpenetrated cubes.

Most of the resulting grains keep the original shape of the Mg(OH)_2_ crystals and exhibit faces that are oriented along the (111) planes. For samples that were treated at *T* = 473 K in vacuum, these faces consist of regular aggregates of interpenetrated cubelets with edges in the 2–5 nm range, as shown via TEM (Figure 6b). The corners of the cube intersections of (100), (010), and (001) terraces are oriented parallel to the plate, i.e., along the [111] direction. The average sizes of these building units grow after vacuum annealing to higher temperatures, i.e., *T* = 1173 K (Figure 6f).

Because of the continuous removal of water from the residual gas atmosphere during simultaneous pumping and annealing, the high surface area of these materials is retained. Even after vacuum annealing to 1173 K, *S*_BET_ values of 236 ± 23 m^2^·g^–1^ are measured (Table 1). The resulting average crystallite domain size is similar to the starting CVS powder’s, indicating that without water and its pronounced effect on the sintering behavior of metal oxide nanostructures, mass transport, and particle coalescence are effectively suppressed.

### Stability of the MgO(111) Surface

**Figure 7:**
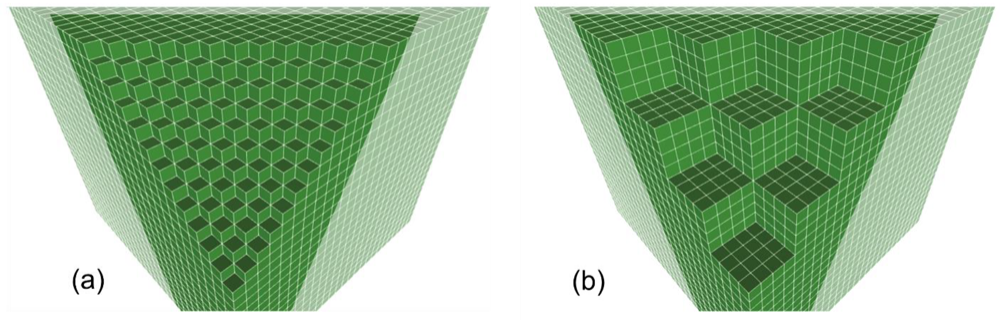
Scheme illustrating the difference between (a) a true (111) surface plane where every corner depicts an individual ion, either O^2–^ or Mg^2+^, or (b) a faceted MgO(111) surface where cube intersections of (100), (010), and (001) planes are oriented parallel to the plate, i.e., along the [111] direction.

Recent publications in materials chemistry report the enhanced reactivity and adsorption capacity of polar metal oxide (111) surfaces (Figure 7a).^19,20,54^ These studies, and other work, have established that the so-called polar (111) surfaces reported by previous groups are not bare (i.e., atomically clean) polar (111) surfaces but are actually stabilized with hydroxyl groups.^55^

Additionally, we provide evidence that vacuum annealing of Mg(OH)_2_ nanostructures at 473 K or 1173 K produces microfaceted surfaces with a high abundance of exposed (100) planes (Figure 7b), most likely due to the thermally induced adsorbate decomposition and surface dehydroxylation.^42^ Therefore, some of the adsorption enhancement effects reported previously^56^ might arise from the decomposition of the (111) surface ligand during the various sample treatments.

To directly probe the stability of the (111) facet, we carried out experiments to remove the stabilizing hydroxide groups from highly characterized MgO(111) nanosheets. The MgO(111) nanosheets probed herein are 200–500 nm in diameter and 3–5 nm thick with a BET surface area of 200(±10) m^2^·g^–1^ determined by nitrogen physisorption analysis. HRTEM studies provided evidence of the (111) MgO surfaces by looking at nanosheets that were parallel to the optic axis of the TEM. Theoretical and experimental results strongly suggest that the polar surface is stabilized by surface hydroxyl groups, which lowers the energy of the unstable (111) outer surface.^14^ In addition, previous work has found the surface to contain highly active corner sites, step terraces, and point defects exposing O^2–^ anions.^47^ Temperature-programmed desorption (TPD) analyses with CO_2_ show that the MgO(111) surface possesses primarily medium basic Mg^2+^ and O^2–^ pairs, followed by surface hydroxyl groups, while both commercial and nanoscale (∼4 nm cubes) samples with the MgO(100) surface have weaker Lewis base sites.^47^

**Figure 8:**
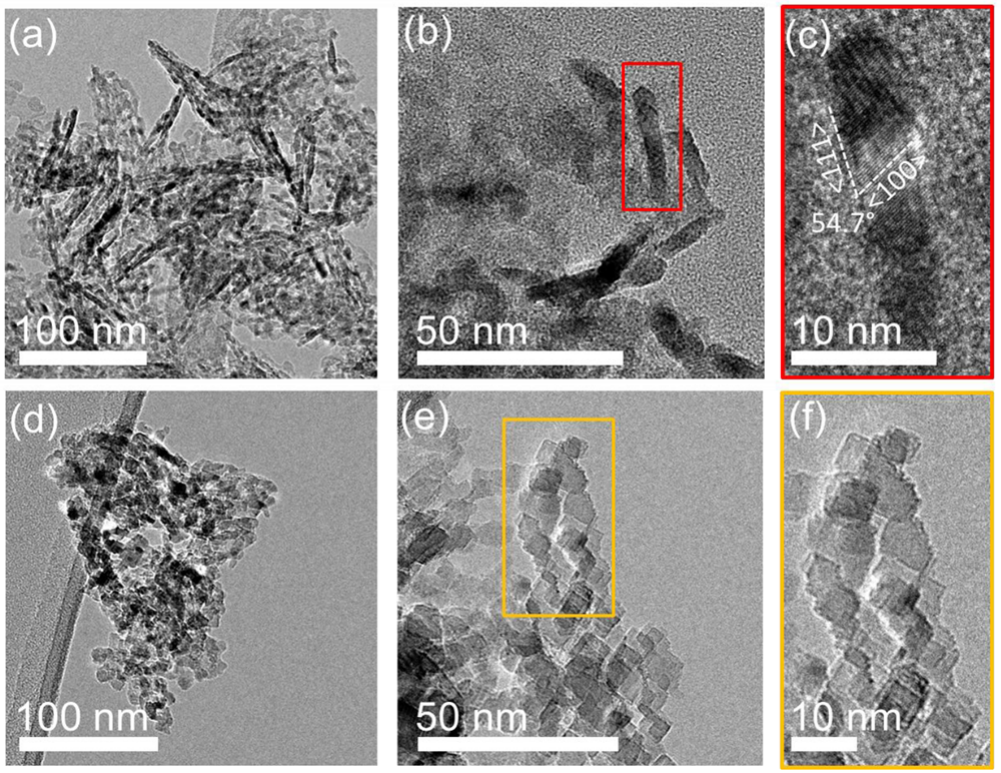
Influence of thermal annealing on MgO (111). (a–f) Bright-field TEM images. (a–c) Pristine MgO(111) nanoparticles. (c) A magnified image of the region outlined by the red square in (b) showing the (111) terminated surface of the rod-like particles. (d–f) MgO(111) after vacuum annealing at 900 °C. (f) A magnified image of the region outlined by the yellow square in (e) showing the reconstruction into MgO(100) terminated cubes.

**Figure 9:**
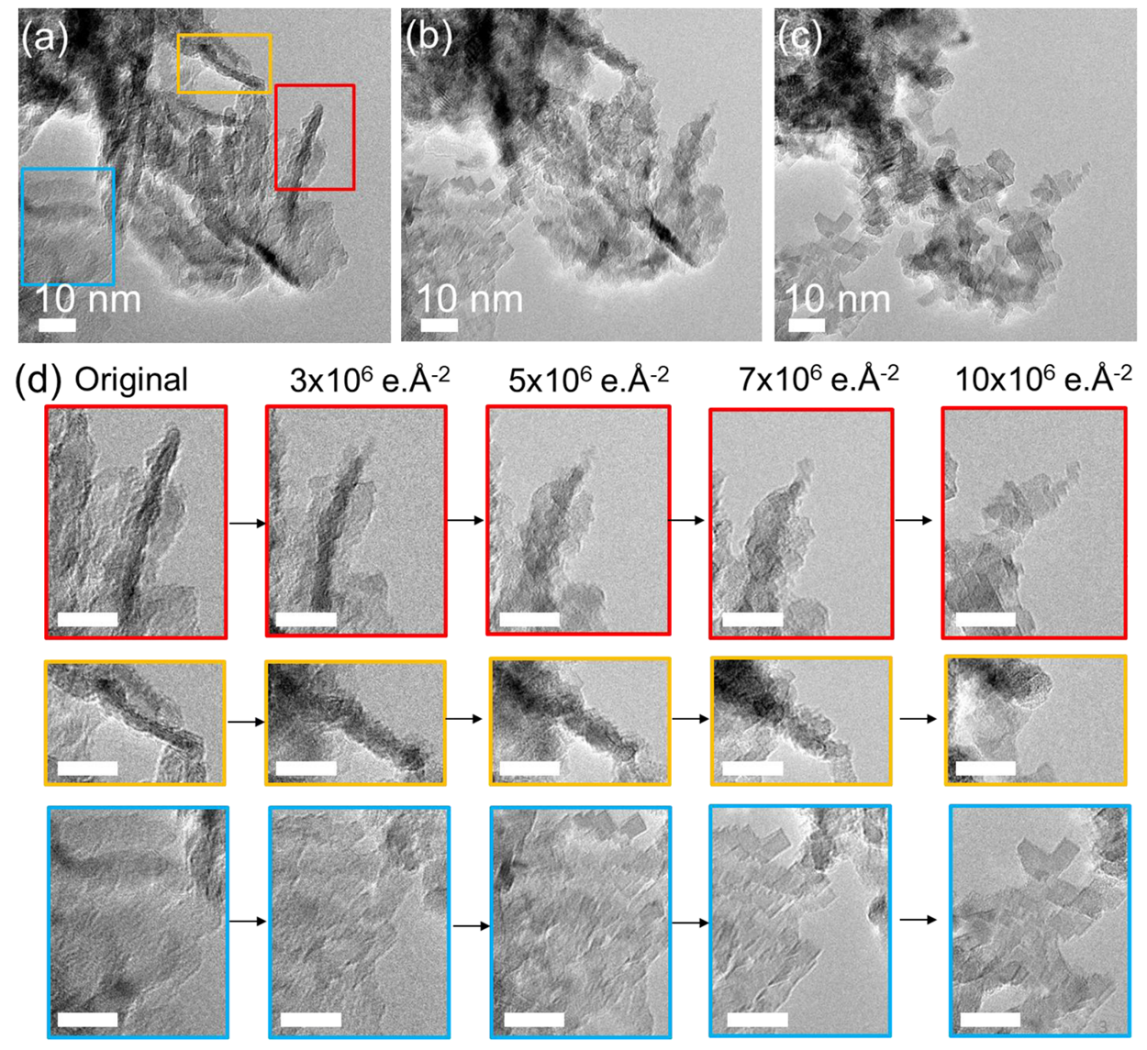
Influence of electron-beam irradiation on MgO(111). (a–d) Bright-field TEM images of MgO (111). (a–c) The same area was imaged: (a) Pristine MgO(111), (b) after a dose of 5 × 10^6^ e·Å^–2^, and (c) after a dose of 10 × 10^6^ e·Å^–2^. (d) Evolution of the three areas outlined by colored squares in (a) as a function of electron dose. Scale bars in (d) 10 nm.

To investigate the stability of bare MgO(111) surfaces, we studied the morphological changes of MgO(111) nanosheets described above under vacuum annealing (Figure 8) and under extended electron-beam irradiation (Figure 9). Vacuum annealing at 1173 K and subsequent exposure to O_2_ (more details in the Experimental Methods) is known to remove all residues from MgO nanoparticles, providing clean and bare MgO surfaces. After such a vacuum annealing, the MgO(111) almost completely converted into MgO(100) nanocubes (Figure 8). This suggests that the removal of the hydroxyl groups from the MgO (111) surfaces in combination with sample exposure to heat leads to its reconstruction into (100) surfaces.^58^

We made a similar observation under sustained electron-beam irradiation during TEM investigation of the pristine (i.e., nonannealed) MgO(111) sample, as seen by the progressive transformation of the MgO(111) surfaces into MgO(100) cubes during imaging (Figure 9). This can be explained by the fact that inelastic scattering of the incident high-energy electrons by the sample can lead to surface reconstruction due to (i) beam-induced heating and (ii) removal of the hydroxyl groups stabilizing the MgO surfaces via knock-on damage (atom sputtering and atom displacement of OH are both expected to occur at 200 kV).^57^ These results taken in conjunction with those above detailing the generation of (111) faceting via topotactic reconstruction of (100) nanocubes emphasizes that interconversion between 100 and 111 faceting is easily controllable, and thus a facile method to tune the activity for specific applications.

## Conclusions

Water adsorption at the solid–gas interface of MgO nanostructures and their subsequent dissolution in gaseous or liquid water can produce 1D and 2D structures, and particles of high morphological definition, with staggered and interpenetrated nanocubes as primary building blocks. This study reports on the different steps involved in these transformations and demonstrates the following:

- H_2_O adsorption-induced organization of MgO nanocubes and their further dissolution–recrystallization into well-defined nanostructures represent an attractive approach to generate extended faceted structures of cubic ionic metal oxide nanoparticles with rock salt structure.
- Mg(OH)_2_ sheet formation and sheet exfoliation in pure water are natural and generic processes that do not require specific counterions or surfactant species.
- Mg(OH)_2_ nanosheets can easily undergo decomposition and faceting to transform into MgO grains and platelets with uniformly shaped grain faces that can be described as inverse cube elements. This topotactic conversion of hydroxide into the metal oxide can be achieved on products of different synthesis approaches and Mg/MgO precursors, such as vapor-phase grown MgO cubes or sol–gel-derived MgO(111) nanosheets (Figures 6 and 8).

The availability of MgO(111) nanosheets as well as MgO-based nanocube arrays, where the cubic building blocks are organized at different levels of order, ranging from randomly oriented particles within dry powders, 1D bars of staggered nanocubes, to reconstructed faceted surfaces of inverse cubes, opens a range of opportunities for adsorption studies and heterogeneous catalysis research.^59^

Furthermore, we show that the continuous desorption of H_2_O molecules from the gas phase through pumping during annealing effectively suppresses particle growth and coarsening. Thus, thermally stable porous microstructures exclusively composed of MgO nanocubes can be synthesized with a variety of configurations without compromising the parent particle size. In addition to the potential of such architectures as substrates for heterogeneous catalysis, our findings are relevant for the development of sintering approaches to manufacture and functionalize nanocrystalline ceramics.

Future theoretical and experimental studies on the reactivity of the inverse cube arrays featuring MgO^8^ planes and thus, a high abundance of edge features with four-coordinated ions, should also encompass cooperative effects between adsorbates and the different surface elements. Moreover, calculations to assess the energetics of the topotactic transformation process (rather than the octopolar reconstruction^16^) in relation to the energy required for the decomposition of the most stable surface hydroxyls would be most useful.

Finally, our results show that MgO(111) nanostructures reconstruct into MgO(100) surfaces upon surface adsorbate removal, suggesting that these MgO(111) surfaces are not bare and thus not polar under surface science experimental conditions. We believe that the important question of how charge-compensating surface groups such as hydroxyls or methoxy groups can enhance and promote the catalytic activity and adsorption capacity as reported for MgO(111) nanosheets^20,35,56^ requires further attention.

## Supporting Information Available

Complementary transmission electron microscopy images and a X-ray diffraction pattern (PDF)
