# Supplementary material for: Cubes to Cubes: Organization of MgO Particles into One-Dimensional and Two-Dimensional Nanostructures
Source: Cryst Growth Des. 2021 Jul 2;21(8):4674–82. doi: 10.1021/acs.cgd.1c00535 (PMC8343528; doi:10.1021/acs.cgd.1c00535)
Supplement: Supplementary file 1 — cg1c00535_si_001.pdf [file cg1c00535_si_001.pdf]

Supporting Information

Cubes to Cubes: Organization of MgO Particles into 1D and 2D Nanostructures

Daniel Thomele[1,2], Stefan O. Baumann[2], Johannes Schneider[1,2], Andreas K. Sternig[2], Sarah Shulda[3], Ryan M. Richards[3], Thomas Schwab[1], Gregor A. Zickler[1], Gilles R. Bourret[1], Oliver Diwald[1*]

[1]Department of Chemistry and Physics of Materials, Paris-Lodron University Salzburg, Jakob Haringerstrasse 2a, Salzburg, 5020, Austria

Email: oliver.diwald@sbg.ac.at

[2]Institute of Particle Technology (LFG), Friedrich-Alexander-Universität Erlangen-Nürnberg, Cauerstraße 4, Erlangen, 91058, Germany

[3]Department of Chemistry, Colorado School of Mines, Golden, CO 80401, United States of

America

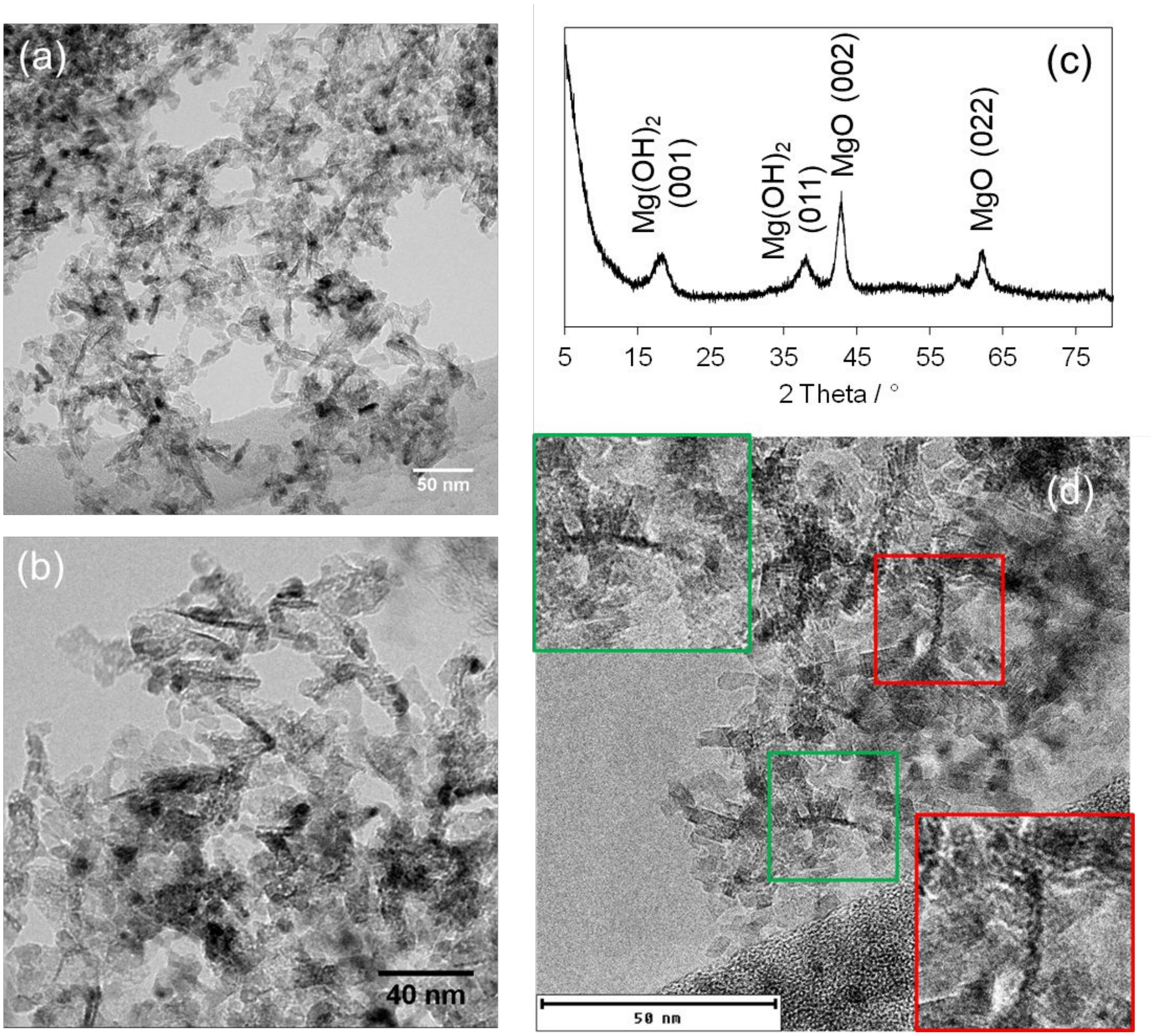

Figure S1: (**a** and **b**) TEM images of MgO nanocube powder samples that were previously exposed to H_2O vapor for 2 hours at $p = 30$ mbar. (c) XRD pattern of corresponding samples reveal 1:1 phase mixture of periclase (MgO) to brucite ($Mg(OH)_2$). Even shorter contact times, such as 10 minutes are sufficient to generate rod-like structures inside a MgO nanoparticle powder (green and red squares in Figure S1d.
